# Improved dynamic contrast-enhanced magnetic resonance angiography (CE-MRA) using iterative data reconstruction

**DOI:** 10.1186/1532-429X-18-S1-O112

**Published:** 2016-01-27

**Authors:** Luigia D'Errico, Michaela Schmidt, Jens Wetzl, Christoph Forman, Aurelien F Stalder, Bernd J Wintersperger

**Affiliations:** 1 Department of Medical Imaging, University of Toronto, Toronto, ON Canada; 2 Siemens Healthcare GmbH, Erlangen, Germany; 3 Department of Computer Science, Friedrich-Alexander-Universität Erlangen-Nürnberg, Erlangen, Germany; 4 Erlangen Graduate School in Advanced Optical Technologies (SAOT), Friedrich-Alexander-Universität Erlangen-Nürnberg, Erlangen, Germany

## Background

CE-MRA following the injection of a gadolinium-based contrast agent (GBCA) bolus is widely used in clinical assessment of vascular dynamics and tissue perfusion (1,2). Although keyhole techniques with an increased number of central k-space updates further improved contrast kinetics information, spatial resolution remains limited. Alternatively applied view sharing approaches also result in prolonged temporal footprints with possible temporal blurring or reconstruction artifacts. Sparse, incoherent sampling approaches and iterative reconstruction techniques have proven successful particularly in dynamic MR imaging (3). We sought to evaluate the impact of iterative reconstruction techniques on dynamic contrast enhanced MRA of the thoracic aorta.

## Methods

Dynamic MRA data sets of 11 patients acquired for thoracic aortic pathology at 3T (MAGNETOM Skyra fit, Siemens Healthcare GmbH) were included. Standard parasagittal TWIST was employed with a slab thickness of 105.6 mm. *K*-Space coverage employed a central *k*-space region (A-15%) and differently sampled peripheral *k*-space regions (B-20%, B1-B5) after bolus injection (3 ml/s) of 2 ml Gadobutrol diluted with 6 ml NaCl. With a spatial resolution of 1.2 × 1.0 × 1.2 mm^3^ (interpolated to 1 mm^3^) and *R* = 4 × 2 GRAPPA acceleration acquisition time for each partial *k*-space (A/B1-B5) was 1.2 s. Standard (TWIST) and prototype iterative algorithm reconstruction (IT-TWIST) was performed on *k*-space raw data. TWIST was followed by accelerated static MRA (GRAPPA *R* = 3) with 0.9 × 0.9. × 1.2 mm^3^ resolution in 22 s acquisition time (32 ml of 1:3 diluted Gadobutrol, 3 ml/s). All TWIST and static MRA were visually assessed (5-point Likert scale) by two blinded readers for contrast-to-noise (CNR), vessel delineation and pulmonary vasculature quality (TWIST/IT-TWIST only). Wilcoxon rank sum test was used for statistical comparison.

## Results

**Figure 1:**
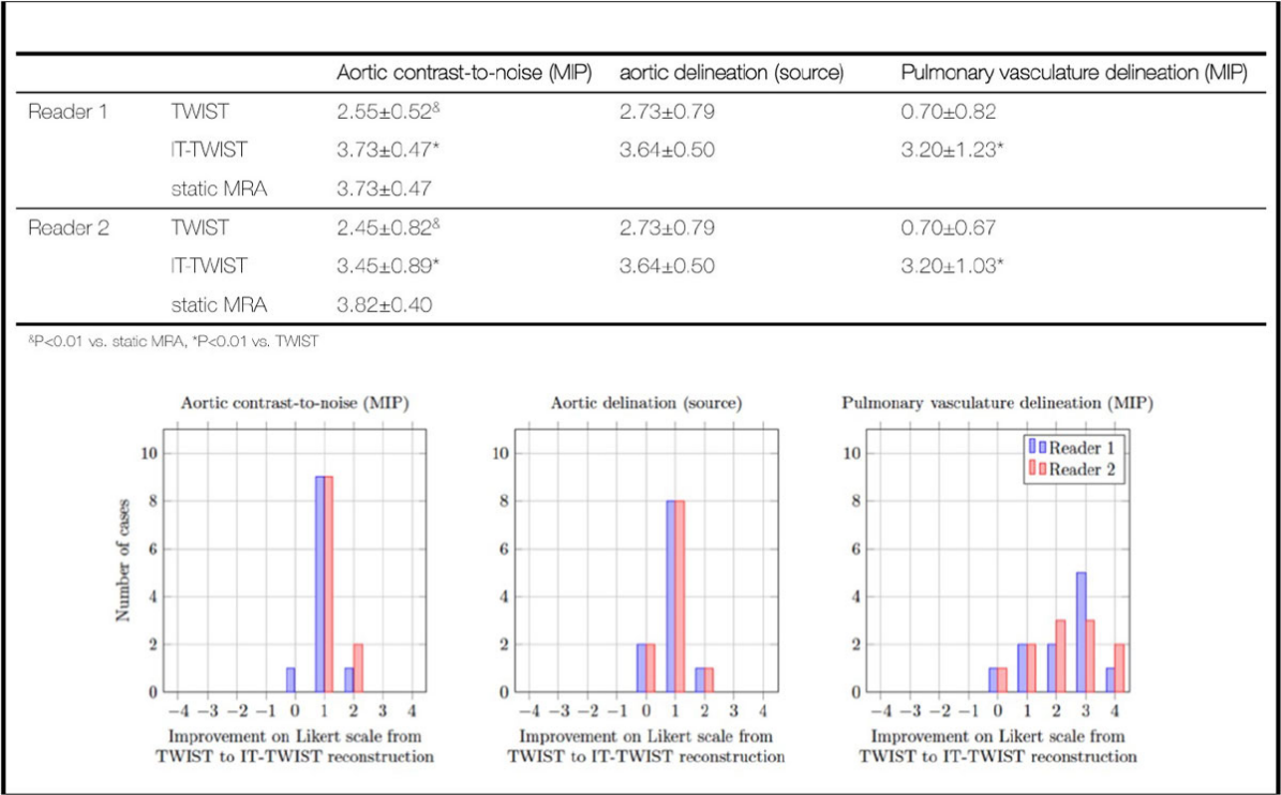
**Mean and standard deviation of image quality ratings for overall appearance of the aortic CNR, large arterial vessel delineation and mid/small size vasculature delineation (top) for TWIST, IT-TWIST and static MRA (aortic CNR only)**. Improvements of these criteria between TWIST and IT-TWIST on a case-by-case basis are shown in the graphs in the bottom row.

**Figure 2:**
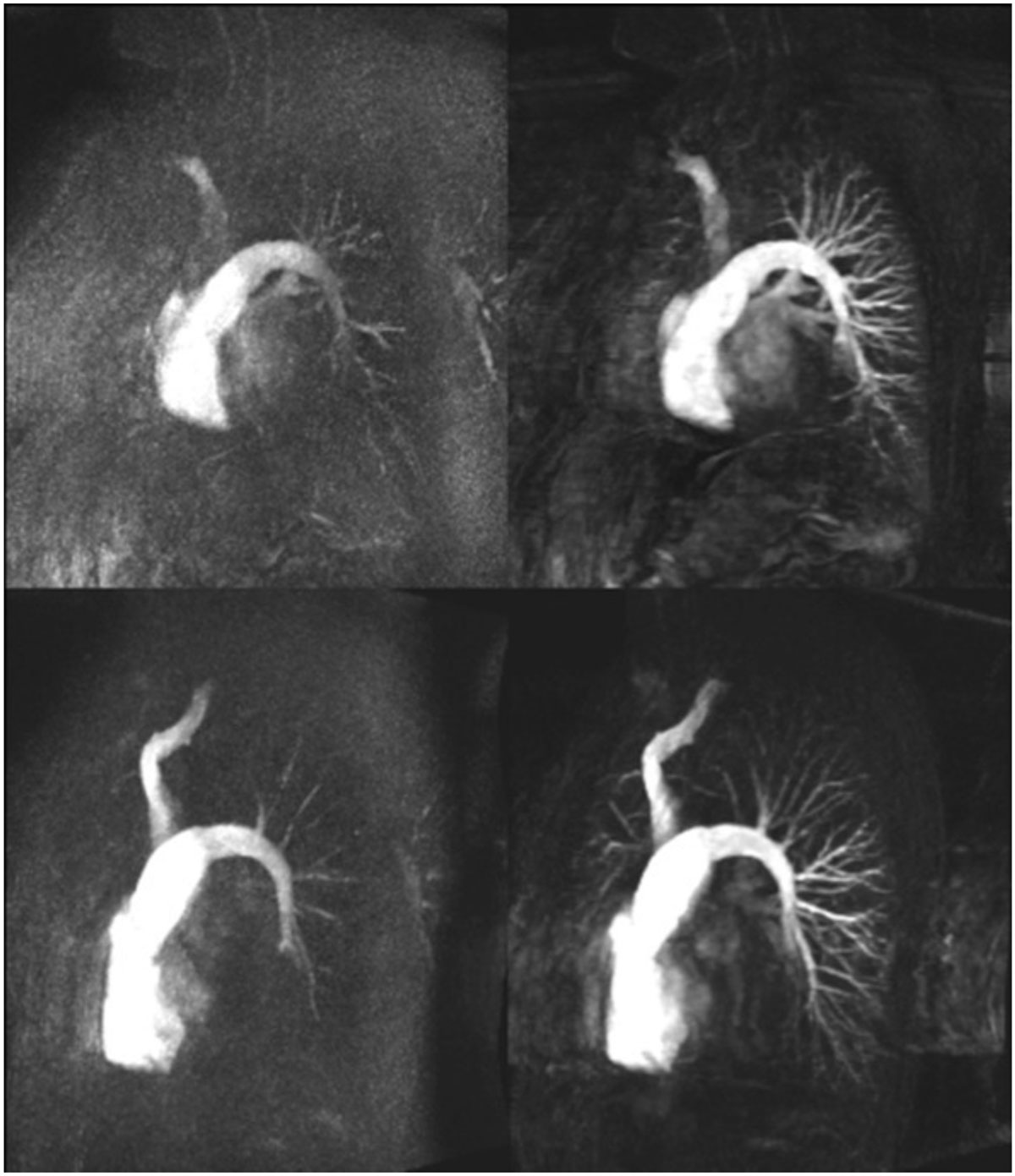
**Comparison of standard and iterative reconstruction MIP time frames in two patients**. In both cases, the identical pulmonary phase of the dynamic CE-MRA demonstrates markedly improved pulmonary vessel delineation for iterative reconstruction (right column) vs. standard reconstruction (left column).

With iterative reconstruction the temporal footprint improved from 10.8 to 2.4 s. With respect to the overall appearance of the aortic CNR, large arterial vessel delineation and mid/small size vasculature delineation, both readers rated IT-TWIST significantly higher than TWIST (Fig 1). Especially for mid/small size vasculature, the improvements were considerable (Fig 2). In comparison to static high-res MRA, iterative reconstruction resulted in non-statistically significant differences for overall aortic CNR while standard reconstruction was significantly inferior. In no case did iterative reconstruction result in worse image quality (Fig 1).

## Conclusions

Iterative reconstruction substantially improves mid/small vessel delineation in dynamic CE-MRA. This combination overcomes the limitation of poor dynamic pulmonary MRA at 3T with standard reconstruction techniques. It has the potential to eliminate the need for bolus timing, to reduce GBCA volume and to eventually replace static MRA.
